# Endometrioid Adenocarcinoma Metastatic to the Thyroid, Presenting Like Anaplastic Thyroid Cancer

**DOI:** 10.1155/2011/246872

**Published:** 2011-08-11

**Authors:** Natasha Pollak, Gregory J. Renner, Ronald Miick, Shellaine R. Frazier

**Affiliations:** ^1^Department of Otolaryngology-Head and Neck Surgery, Temple University School of Medicine, Kresge West, 3rd floor, 3400 North Broad Street, Philadelphia, PA 19140, USA; ^2^Department of Otolaryngology-Head and Neck Surgery, University of Missouri-Columbia, Columbia, MO 65212, USA; ^3^Department of Pathology and Laboratory Medicine, Albert Einstein Medical Center, Philadelphia, PA 19141, USA; ^4^Department of Pathology and Anatomical Sciences, University of Missouri-Columbia, Columbia, MO 65212, USA

## Abstract

Metastasis of uterine cancer to the head and neck is extremely rare. We report what we believe to be the first documented case of endometrioid adenocarcinoma metastasizing to the thyroid gland. An 80-year-old woman was referred to the otolaryngology service with a rapidly growing neck mass. The mass appeared to originate from the thyroid gland. Her clinical presentation was consistent with anaplastic thyroid carcinoma. A tracheostomy was performed. An open biopsy established the diagnosis of moderately differentiated adenocarcinoma, consistent with a gynecologic primary. The patient had undergone a hysterectomy 5 years prior for endometrioid adenocarcinoma. The thyroid tumor histology and immunophenotype corresponded well with her prior endometrial carcinoma, indicating that the thyroid mass was a metastasis from the endometrial primary. Radiotherapy appears to offer good local disease control in this rare case of endometrioid adenocarcinoma metastatic to the thyroid.

## 1. Introduction

Endometrioid adenocarcinoma is a fairly common uterine malignancy with an incidence of 24.4 cases per 100,000 in the United States. This paper describes the first known case of metastasis of endometrioid adenocarcinoma to the thyroid gland. The patient's clinical presentation mimicked anaplastic thyroid cancer. We discuss initial management, diagnosis, and long-term treatment with radiation therapy.

## 2. Case Presentation

An 80-year-old woman was referred to the otolaryngology service with a three week history of a large, rapidly growing mass in the low right anterior neck. A fine-needle aspiration biopsy reported large cell carcinoma. A contrast-enhanced CT scan of the neck identified a 7 cm complex mass in the region of the right thyroid lobe. The mass was displacing the airway to the left. No cervical adenopathy was seen. Her clinical presentation was consistent with anaplastic thyroid carcinoma. In view of the rapid growth and progressing symptoms of dysphagia and airway obstruction, a tracheostomy was planned. Partial tumor debulking was necessary in order to place the tracheostomy. Intraoperatively, the mass appeared to originate from and involve the right thyroid lobe. An open biopsy established the diagnosis of moderately differentiated adenocarcinoma. Five years prior, the patient was diagnosed with endometrioid adenocarcinoma on biopsy. She underwent a total abdominal hysterectomy and bilateral salpingo-oophorectomy, which confirmed a FIGO grade II endometrioid adenocarcinoma. The patient was subsequently treated with high-dose radiation therapy (HDR) to the vaginal cuff, 1800 cGy × 3. She was free of recurrence or metastases up to this point.

**Figure 1:**
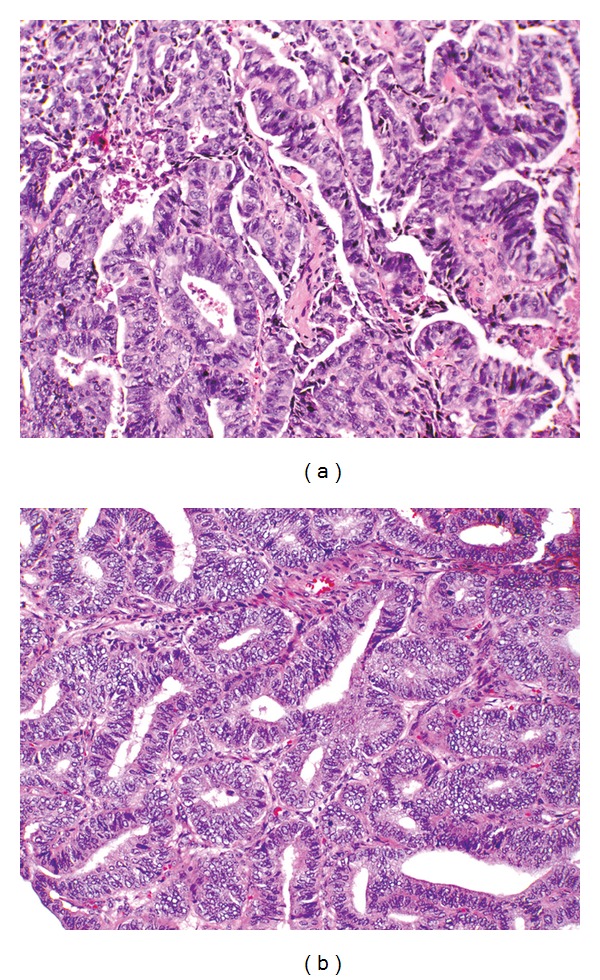
Thyroid mass composed of branching glands with columnar cells showing nuclear pseudostratification and atypia with scattered mitoses (a). Comparison with the patient's hysterectomy specimen (b) reveals a similar morphology.

**Figure 2:**
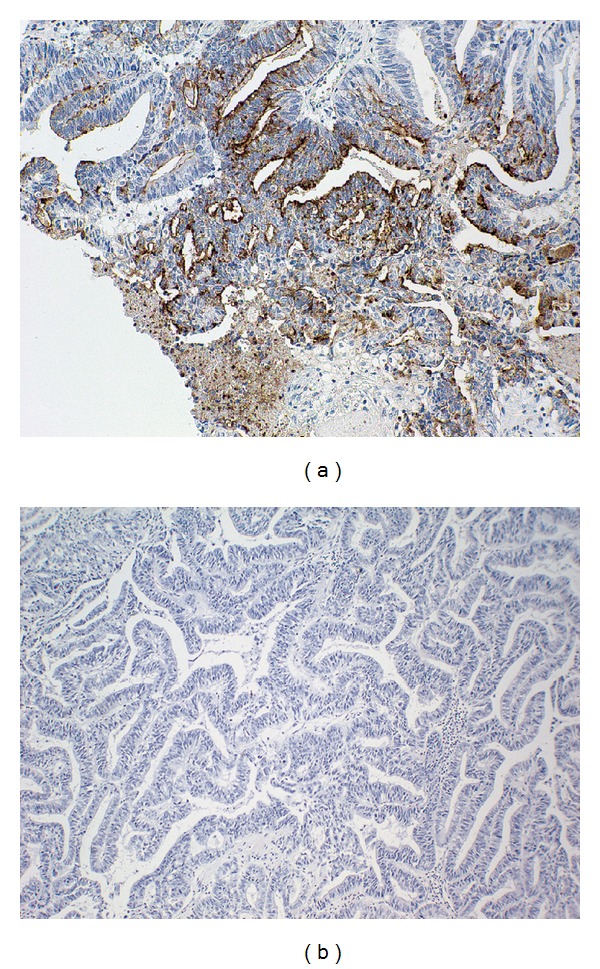
Thyroid mass with positive immunoreactivity for CA-125 (a) but no immunoreactivity for thyroglobulin (b).

Sections of the thyroid mass show a malignant neoplasm forming glands in a necrotic background comprised of columnar cells with large hyperchromatic pseudostratified nuclei, prominent nucleoli, and atypia. Immunohistochemical stains are negative for thyroglobulin, thyroid transcription factor-1 (TTF-1), and calcitonin. A comparison of the thyroid mass with the patient's endometrioid adenocarcinoma (FIGO Grade II) diagnosed five years earlier, reveals a similar histology. (Figure 1) A CA-125 immunostain is positive in the thyroid tumor, consistent with a metastasis from a gynecologic primary (Figure 2).

## 3. Discussion

Metastasis of uterine cancer to the head and neck is extremely rare. We report what we believe to be the first documented case of endometrioid adenocarcinoma metastasizing to the thyroid gland as the first presentation of any regional or distant disease. Endometrioid adenocarcinoma normally spreads to the pelvic and paraaortic lymph nodes and ovaries. Most frequently, distant metastases have been reported to the lung, liver, and bone. Rare case reports can be found in the literature of endometrioid carcinoma metastases to the head and neck region, including the soft tissues of the neck [1], paranasal sinuses [2], eye [3], brain [4], tongue [5], skin [6], and maxilla [7]. A single case of an endometrial carcinosarcoma metastatic to the thyroid has also been reported [8]. 

 The histologic differential diagnosis includes the columnar cell variant of papillary thyroid carcinoma (PTC), anaplastic thyroid carcinoma, and metastasis. Columnar cell PTC is excluded on the basis of immunohistochemistry (negative thyroglobulin and TTF-1 staining) as well as histology (i.e., lack of characteristic nuclear morphology such as pseudoinclusions, grooves, and optically clear nuclei). While anaplastic (undifferentiated) thyroid carcinoma is usually negative for TTF-1 and thyroglobulin, the histomorphology of the mass shows neither dedifferentiated areas nor foci of PTC, making anaplastic thyroid carcinoma unlikely. While thyroid carcinomas (all types) can be positive for CA-125 in up to 10% of cases [9], endometrioid carcinomas are positive for CA-125 in up to 91% of cases [9, 10]. Ultimately, a comparison with the patient's previous uterine carcinoma, along with CA-125 positivity, confirms the diagnosis of metastatic endometrioid adenocarcinoma to the thyroid. 

 The importance of this case lies in the fact that, on initial presentation, it mimicked the rapid growth of anaplastic thyroid carcinoma. The fine-needle aspirate was not helpful in this case. Open biopsy was necessary in order to establish the correct diagnosis. The patient was treated with radiation therapy to the neck, and was without evidence of disease six months after treatment.

 This paper broadens the differential diagnosis of rapidly enlarging thyroid masses to include metastasis from gynecologic primary malignancies. So far, radiation therapy appears to offer good local disease control in this rare case of endometrioid adenocarcinoma metastatic to the thyroid gland.
